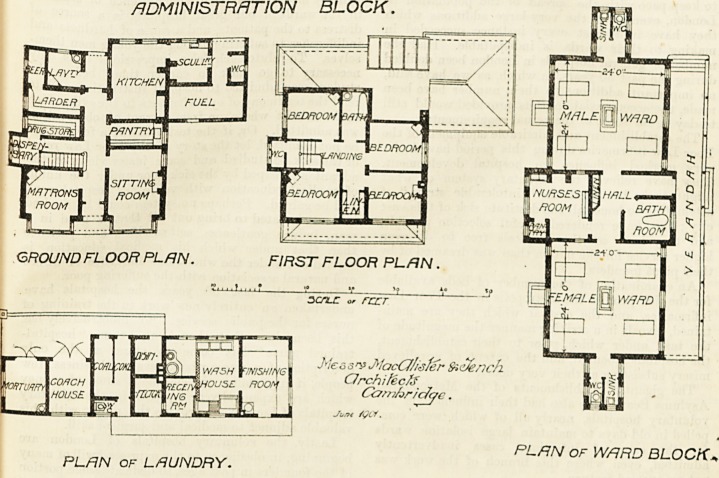# Isolation Hospital, Royston

**Published:** 1901-10-05

**Authors:** 


### ISOLATION HOSPITAL, ROYSTON.

A new isolation hospital is being erected at Royston in Hertfordshire for the Joint Hospital Board of the district. It consists of a ward block for eight beds, an administrative block, a laundry block, and a caretaker's lodge. The hospital proper consists of a four-bedded ward for men, a similar one for women, a centre containing bath-room, hall, nurses' duty-room, and linen store. Each ward is 24 feet square, and assuming that it is 14 feet high, the cubic space per bed will be a little over 2,000 feet. The sanitary arrangements are good, and a verandah runs along the whole length of the block. The administrative department is compact, but there is no special feature in it calling for remark. The laundry is also well arranged.

The hospital will be warmed by ventilating stoves, and the floors will be covered with papyrolith. The drainage will be treated on the septic tank system. The architects are Messrs. MacAlister and Tench of Cambridge. The estimated expense is £3,500.

## Figures and Tables

**Figure f1:**